# Prevalence and predictors of irritable bowel syndrome among medical students and interns in King Abdulaziz University, Jeddah

**DOI:** 10.3402/ljm.v8i0.21287

**Published:** 2013-09-19

**Authors:** Nahla Khamis Ragab Ibrahim, Wijdan Fahad Battarjee, Samia Ahmed Almehmadi

**Affiliations:** 1 Family and Community Medicine Department, Faculty of Medicine, King Abdulaziz University, Jeddah, Saudi Arabia; 2 High Institute of Public Health, Alexandria University, Alexandria, Egypt; 3 King Abdulaziz University, Rabigh Branch, Jeddah, Saudi Arabia; 4 Department of Psychiatry, Al-Amal Hospital, Jeddah, Saudi Arabia

**Keywords:** epidemiology, irritable bowel syndrome, medical students, Jeddah

## Abstract

**Background:**

Irritable bowel syndrome (IBS) is a frequent, costly, and potentially disabling gastrointestinal disorder. Medical education is among the most challenging and the most stressful education, and this may predispose to high rates of IBS.

**Objective:**

To determine the prevalence and predictors of IBS among medical students and interns in King Abdulaziz University, Jeddah, Saudi Arabia.

**Methods:**

A cross-sectional study was conducted among 597 medical students and interns selected by multistage stratified random sample method in 2012. A confidential, anonymous, and self-administered questionnaire was used to collect personal and sociodemographic data, level of emotional stress, and food hypersensitivity during the past 6 months. Rome III Criteria and the Standardized Hospital Anxiety and Depression Scale were also used.

**Results:**

The prevalence of IBS was 31.8%. Multiple logistic regression analysis revealed that the first predictor of IBS was female gender (aOR=2.89; 95.0% CI: 1.65–5.05). The second predictor was presence of morbid anxiety (aOR=2.44; 95.0% CI: 1.30–4.55). Living in a school dormitory, emotional stress during 6 months preceding the study, and the academic year were the next predictors.

**Conclusions:**

High prevalence of IBS prevailed among medical students and interns. Female gender, morbid anxiety, living in school dormitory, emotional stress, and higher educational level (grade) were the predictors of IBS. Screening of medical students for IBS, psychological problems, and reducing stress by stress management are recommended.

There is a growing concern toward clinical and epidemiological research in the area of functional gastrointestinal disorders (FGIDs) (1). Irritable bowel syndrome (IBS) is a common, costly, and potentially disabling FGID. It is characterized by abdominal pain or discomfort with changed bowel habits but without any organic damage to the intestine (tumor or inflammation) (2–4). The etiology of IBS is uncertain, and studies have documented that psychological, social, and biological factors can play a role (5).

IBS creates an incredible cost for both patients and the health care system (6–9). It is one of the commonest disorders diagnosed by gastroenterologists (8, 9). There is a large part of the population suffering from IBS while only some seek health care in the absence of curative therapy (6, 7, 9, 10). The prevalence of IBS usually varies significantly between countries and depends on the diagnostic criteria used (11). A study conducted among secondary school male students in Al-Jouf Province, Saudi Arabia, showed that the prevalence was 8.9 and 9.2% according to Manning and Rome II Criteria, respectively (12).

Regarding university students, the prevalence of IBS was found to be 5.7% in one of the Korean colleges (2). Medical students are under constant stress; the duration it takes to complete their studies, numerous exams, difficult shifts, and the responsibility of managing patients may cause much stress. The role of stress can partly justify the high prevalence of IBS seen among medical students (6, 11, 13, 14). In 2012, Chu et al. conducted a study among medical, science, and engineering students in China . They reported that medical students had a much higher risk of functional bowel disorders (FBD) than science and engineering students (15). Another study conducted in 2012 among medical students at the University of Western Ontario, Canada (16), found that the prevalence of IBS among preclinical and clerkship students was 19.1 and 22.0%, respectively.

Although IBS is a widespread functional disorder in Western countries, little is known about its prevalence in Arab countries, including Saudi Arabia. In addition, information on the prevalence and the associated factors among university students, especially medical students, is relatively scant (17). In addition a limited number of studies have been conducted among medical students in Jeddah. As a result, such an epidemiological study among medical students is needed.

The present study aimed at determining the prevalence and predictors of IBS among medical students and interns at King Abdulaziz University (KAU) in Jeddah, Saudi Arabia.

## Materials and methods

A cross-sectional study was conducted in KAU during 2011–2012. The study population included students from the second to the sixth year of medical school, and interns. A multistage stratified random sample method was used where the total size of the population was sampled. Stratification took into consideration gender and educational level. The sample was first stratified into male and female with a ratio of 1:1. The second stratification was according to grade from second to sixth year. The sample size was calculated according to the following established formula for determination of sample size (18):

$$n=\frac{z^{2}\times p\times q}{d^{2}}$$

*n*=the minimum sample size, *z*=constant (1.96). We assumed a prevalence of 14% on the basis of a previous study (8); p=0.14 and q=1−p=0.86. The minimum calculated sample size to achieve a precision of ±3% with a 95% Confidence Interval (CI) was 514. For stratification purposes, the sample size was increased to 597 during the fieldwork for inclusion of all strata as the size of the sample in each stratum was determined by the proportional allocation method.

The study was approved by the Institutional Review Board of the Faculty of Medicine, KAU Hospital and conformed to the ethical standards of the Helsinki declaration. Administrative approvals were taken. During fieldwork, the objectives of the research were discussed with each participant separately and upon acceptance to participate, consent was taken from each one. A validated, preconstructed, anonymous, confidential, and self-administered questionnaire was used. Reliability was assessed with Cronbach's α and was found to be 85%.

## The questionnaire contained the following sections:

1. Personal and sociodemographic data, such as age, sex, and so on.
2. Family history of IBS.
3. Previous diagnosis of IBS by a physician, and absenteeism from the university due to IBS, if applicable.
4. Other chronic medical conditions.
5. Lifestyle: number of sleeping hours, smoking, exercise, and so on.
6. Traveler's diarrhea and if its presence triggered the first onset of IBS.
7. Emotional stress in the 6-month period preceding the study: loss of a family member, close friend, and so on.
8. Hospital Anxiety and Depression Scale (HADS) (19): It is a standardized, valid, and reliable self-report rating scale. It consists of 14 items: seven for anxiety (HADS-Anxiety) and seven for depression (HADS-Depression). It was answered using a 4-point Likert scale ranging from 0 (not present) to 3 (considerable).
9. Food Frequency Questionnaire (FFQ): a retrospective method of dietary assessment for inquiring about frequency of use of different food items such as milk, yogurt, low fat food, fish, and so on.
10. Rome III Criteria (20, 21): IBS diagnosis was based upon the English version of ‘Rome III Criteria’. Rome III Criteria can rationally diagnose IBS in non-existence of red flag symptoms. The sensitivity of Rome III Criteria in the absence of red flag symptoms is 65%, specificity is 100%, the positive predictive value is 100%, and the negative predictive value is 76% (20).
  IBS was defined according to Rome III criteria as recurrent abdominal pain or discomfort for at least 3 days per month during the past 3 months, associated with two or more of the following features (16): (a) improvement with defecation; and/or (b) onset associated with a change in frequency of stool; and/or (c) onset associated with a change in form (appearance) of stool.
11. Red-flag items: There are seven red-flag items according to the American Gastroenterological Association. Participants reporting ≥1 of these red-flags were excluded from the study (2).
12. Weight and height were measured.

## Statistical analysis

Data were analyzed using Statistical Package of Social Sciences (SPSS) Version 16 (SPSS Inc., Chicago, IL) and Epi-Info Statistical Packages.

Body mass index (BMI) was calculated and divided into: normal, BMI <25; overweight, BMI 25–30; obese, BMI 30–35, and morbidly obese, BMI ≥35.

The total score of HADS is the sum of the 14 items, and for each subscale (anxiety and depression) the score is the summation of the particular seven items (ranging from 0 to 21) (19). Then it is divided into: normal, 0–7; borderline abnormal, 8–10; and abnormal, 11–21 (19).

Descriptive statistics and inferential statistics were carried out. Pearson's Chi-square (*X* ^*2*^) test was conducted to observe and quantify an association between the categorical outcome and the different variables. Odds ratio (OR) with a 95% CI was calculated using Epi-Info. Stepwise multiple logistic regression analysis was applied to delineate significant predictors of IBS among participants. All calculated *P*-values were two-tailed, with *p<*0.05 considered as statistically significant.

## Results

The mean age of medical students and interns who participated in the study was 21.68±1.77 years. The majority of the participants were single (91%), living in their family home (81.4%), and with enough family income (72.0%). Fathers of about three-quarters (75.5%) of the students had a university degree or greater, and fathers of 50.6% of students had a professional job.

Regarding lifestyle and health condition, about one-tenth (10.6%) of participants were smokers and less than one-half (47.4%) practiced physical exercise. The majority of medical students and interns (82.1%) did not report any food hypersensitivity. Most of the participants (82.9%) did not use regular medication.

About one-third (33.8%) of participants reported a family history of IBS, while 14.7% of the total sample reported that they were previously diagnosed by physicians as having IBS. About one-tenth of medical students and interns (10.7%) stated that IBS resulted in their absenteeism from university.

As regards anxiety, analysis of results using HADS-Anxiety revealed that 32.9, 31.1, and 36.1% of the participants had no anxiety, borderline anxiety, and morbid anxiety, respectively. On the contrary, the corresponding rates for depression were 57.5, 27.1 and 15.4%, respectively.

In the current analysis, Rome III Criteria identified 190 students diagnosed as having IBS; giving an overall IBS prevalence of 31.8% among medical students and interns. Only 49 (25.8%) of the total 190 cases were previously diagnosed by a physician.

Table 1 shows that the prevalence of IBS was significantly much higher among females (41.8%) compared to males (22%). IBS was also significantly higher among students aged ≥22 years compared to younger students (*X* ^*2*^ =10.3, *p<*0.001). Prevalence of IBS aggravated with the increase in the academic year (except for the fourth year). The education and occupation of parents did not play a vital role with regard to IBS. On the contrary, students living with their families had significantly lower prevalence of IBS compared to those living in school dormitories. Table 1 also illustrates that married students had a higher prevalence (40.2%) of IBS compared to those who were single (30.9%). However, there is no statistical significant difference (*p*>0.05).

**Table 1:** Relationship between irritable bowel syndrome and personal, sociodemographic characteristics of medical students and interns in King Abdul-Aziz University 2012

Irritable bowel syndrome variable	IBS (No.%)		NO IBS (No.%)		*X* ^*2*^	*p*	OR	95% CI
Sex								
Female	124	41.8	173	58.2	26.83	0.000*	2.54	1.75–3.69
Male	66	22.0	234	78.0				
Age								
<22	42	22.7	143	77.3	10.3	0.001*	0.52	0.35–0.78
≥22	148	35.9	264	64.1				
Academic year								
2nd^(^RC^)^	20	19.8	81	80.2	*30.08*	*0.000* *	*1*	
3rd	27	27.3	72	72.7			1.52	0.75–3.1
4th	21	21.2	78	78.8			1.09	0.52–2.29
5th	31	31.6	67	68.4			1.87	0.93–3.77
6th	45	45.0	55	55.0			3.31	1.69–6.53
Interns	46	46.0	54	54.0			3.45	1.76–6.8
Marital status								
Single	168	30.9	375	69.1	2.18	0.14	0.65	0.73–1.15
Married	22	40.2	32	59.3				
Father education								
Less than university	46	31.5	100	68.5	0.009	0.924	0.98	0.65–1.47
University or above	144	31.9	307	68.1				
Mother education								
Less than university	78	32.4	163	67.6	*0.054*	*0.816*	1.04	0.73–1.48
University or above	112	31.5	244	68.5				
Father occupation								
Professional	102	33.8	200	66.2	1.07	0.3	1.20	0.85–1.69
Non-professional	88	29.8	207	70.2				
Mother occupation								
Professional	68	30.8	153	69.2	0.18	0.6	0.93	0.65–1.32
Non-professional	122	32.8	254	67.6				
Living condition								
With family	152	31.3	334	68.7	9.378	0.009*	***1***	
Private house	18	24.7	55	75.3			0.72	0.39–1.31
School dormitory	20	52.6	18	47.4			2.44	1.20–4.99
Income								
Enough and exceeds	121	28.1	309	71.9	9.63	0.002*	0.55	0.38–0.8
Enough only or not enough	69	41.3	98	58.7				
Parents								
Living together	180	31.4	393	86.6	1.11	0.2	1.56	0.68–3.58
Divorced	10	41.7	14	58.3				

RC Referent catagory

* Statistical significant difference

Table 2 illustrates that students who practised physical exercise had a significantly lower prevalence of IBS than others (OR=0.59; 95% CI: 0.42–0.85). Morbidly obese students and students who slept less hours per day (<8 h/day) had a higher prevalence of IBS compared to other students (*p*<0.05). Similarly, those who obtained higher Grade Point Average (GPA) had slightly higher rates of IBS compared to others. Smoking was not associated with IBS; 33.3% of smokers had IBS compared to 31.8% of non-smokers (*p*>0.05).

**Table 2:** Relationship between irritable bowel syndrome and habits, and health background of medical students and interns in King Abdulaziz University, 2012

Irritable bowel syndrome variable	IBS (No.%)		NO IBS (No.%)		*X* ^*2*^	*p*	OR	CI
Nutritional status (BMI)▴								
Normal^(^RC^)^	112	34.1	216	65.9	8.079	0.04*	1	1
Overweight	33	26.0	94	74	0.68	0.42–1.10
Obese	13	25.5	38	74.5	0.66	0.75–1.22
Morbidly obese	12	52.2	11	47.8	2.1	0.84–5.31
Exercise								
Yes	74	26.1	209	73.9	8.468	0.004*	0.59	0.42–0.85
No	116	37.3	195	62.7
Sleeping hours								
<8 h/day	121	34.3	232	65.7	2.39	0.12	1.32	0.93–1.88
≥8 h/day	69	28.3	175	71.7
Smoking								
Yes	21	33.3	42	66.7	2.29	0.13	1.39	0.90–2.15
No	169	31.8	362	68.2
GPA•								
<4.5	48	25.8	138	74.2	7.94	0.005*	0.56	0.38–0.84
≥4.5	115	38.2	186	61.8

▴ Out of 597 students, weight or height of 68 students was not measured and their BMI was not calculated.

• Out of 597 students, 110 refused to give their GPA.

RC Referent category.

* Statistical significant difference.

It is apparent from Table 3 that the prevalence of IBS was much higher (44.1%) among students with a positive family history of the disease, compared to others (25.6%). IBS was present in a significantly (*p*<0.05) higher rate among students who had food hypersensitivity (64.0%) compared to students without food sensitivity (30.4%). The rate of IBS was higher among participants who had traveler's diarrhea (40.8%) compared to others (30.8%). There is no statistically significant difference between the presence of other chronic diseases and IBS (*p*>0.05).

**Table 3:** Relationship between irritable bowel syndrome and family history, health background of medical students and interns in King Abdulaziz University, 2012

Irritable bowel syndrome variable	IBS (No.%)		NO IBS (No.%)		*X* ^*2*^	*p*	OR	CI
Family history of IBS								
Yes	89	44.1	113	55.9	21.06	0.000*	2.29	1.60–3.20
No	101	25.6	294	74.4
Chronic health problems								
Yes	41	38	67	62.0	2.29	0.13	1.39	0.90–2.15
No	149	30.5	340	69.5
Medication use								
Yes	40	43.7	52	56.3	6.15	0.01*	1.77	1.10–2.86
No	150	30.3	345	69.7
Traveler's diarrhea								
Yes	31	40.8	45	59.2	3.23	0.07	1.57	0.96–2.57
No	159	30.5	362	69.5
Food hypersensitivity								
Yes	16	64.0	9	36.0	12.45	0.000*	4.07	1.76–9.38
No	174	30.4	398	69.6

* Statistical significant difference.

Analysis of different dietary intakes using the FFQ demonstrated that there was no statistically significant difference between intake of different food items and IBS.

Table 4 portrays the relationship between IBS and the psychological aspect of participants; 40.1% of medical students and interns who experienced emotional stress in the 6-months that preceded the study had IBS, compared to only 20.1% among those who did not have such stress (*p<*0.001). After analysis of HADS, the table also revealed that there was a significantly (*p*<0.000) higher prevalence of IBS among participants who had an anxiety problem (prevalence of IBS was 41.6, 31.0 and 23.9% among students with morbid anxiety, borderline anxiety, or no-anxiety, respectively). As for depression, students diagnosed as having morbid depression had a higher prevalence (41.9%) of IBS compared to those with borderline depression (29.5%) or students hadn't depression (31.5%). However, there was no statistical significant difference (*p*>0.05).

**Table 4:** Relationship between irritable bowel syndrome and psychological aspect of medical students and interns in King Abdulaziz University, 2012

Irritable bowel syndrome variable	IBS (No.%)		NO IBS (No.%)		*X* ^*2*^	*p*	OR	CI
Emotional stress in past 6 months								
Yes	139	40.1	208	59.9	25.88	0.000*	2.61	1.79–3.79
No	51	20.4	199	79.6
Anxiety grade•								
Normal^(^RC^)^	44	23.9	140	76.1			1	
Borderline	54	31	120	69	13.95	0.001*	1.43	0.87–2.15
Morbid anxiety	84	41.6	118	58.4			2.27	1.43–3.60
Depression grade▴								
Normal^(^RC^)^	101	31.5	220	68.5			1	
Borderline	44	29.1	107	70.9	4.373	0.112	0.9	0.57–1.40
Morbid	36	41.9	50	58.1			1.57	0.93–2.63

• Out of 597 students, 37 students did not complete HADS-Anxiety.

▴ Out of 597 students, 39 students HADS-Depression.

RC Referent category.

* Statistical significant difference.

Controlling confounding factors in multiple logistic regression analysis revealed that the first predictor of IBS was gender. Females were about three times more likely to develop IBS compared to males (aOR=2.89; 95.0% CI: 1.65–5.05). The second predictor was the presence of morbid anxiety; those diagnosed as having morbid anxiety were 2.44 times more likely to have IBS compared to others (aOR=2.44; 95.0% CI: 1.30–4.55). Those living with their families were less likely to develop IBS compared to the rest (aOR=0.48; 95.0% CI: 0.31–0.75). The last predictor was the academic year; students in their second year were less likely to have IBS compared to other years (aOR=0.77; 95.0% CI: 0.62–0.95; see Table 5).

**Table 5:** Logistic regression analysis of predictors of Irritable Bowel Syndrome among medical students in King Abdulaziz University

Variable	Beta	*p*	aOR	95.0% CI
Gender (female)	1.061	0.000	2.89	1.65–5.05
Anxiety				
Morbid anxiety	0.891	0.005	2.44	1.30–4.55
Borderline anxiety	0.328	0.275	1.39	0.77–2.50
No anxiety^(RC)^		1		
Living condition (with family)	0.736	0.001	0.48	0.31–0.75
Emotional stress	0.620	0.028	1.86	1.07–3.22
Academic year (second year)	0.261	0.018	0.77	0.62–0.95
Constant	−1.428			

RC Referent category.

## Discussion

The prevalence of IBS varied greatly among different investigations. The range of IBS prevalence was 15%–24% among the general population of Western countries (2). Hungin et al. (2003) conducted an international study among 41,984 individuals across eight European countries and found that the prevalence of IBS was 11.5% (22). The current study illustrates a higher prevalence of IBS (31.8%) among medical students and interns. The discrepancy between the current study and the European study suggests that there might be a true difference between countries. It may be of interest in the future to attempt to correlate these differences with the cultural and dietary habits in various countries (22). Other causes may be attributed to sample size, age group, and diagnostic criteria used.

Regarding different types of students, Chu et al. reported that medical students had a higher risk of any FBD than science and engineering students (15). Results from Japan showed that the rate of IBS was 35.5% among nursing and medical students (13). Similar rates were reported among medical students from two Pakistani studies; 28.3% in 2012 (20) and 34% in 2005 (9). The rate was 29.2% among medical and paramedical students from Korea (4). These rates are in line with the results of the current study.

On the contrary, a lower prevalence (15.8%) than that of the current study was reported from an earlier study conducted by Tan et al. in 2003, among young multi-ethnic medical students from Malaysia, using Rome I Criteria (23). Shen et al. (17) reported a similar prevalence among Chinese university students. The cause of a discrepancy between the current study and the previous studies may, in part, be related to ethnic differences in IBS frequency. These differences could be attributed to genetic or environmental factors or a combination of both (24).

In 2003, a study from the United States reported that only 11% of the college students met the criteria of IBS (25). Alhazmi et al. 2011 (12) conducted a study among Saudi secondary school students and reported that the prevalence was 8.9 and 9.2% according to Manning and Rome II Criteria, respectively. The inconsistency between those two previous studies and our study may be due to the enormous stress of medical student life, which predispose students to IBS (6, 13). The length of time it takes to complete medical studies, the numerous difficult exams, and the irregular working hours are examples of such stress (6). Jimenez et al. 2010 (14) identified three stressors (clinical, academic, and external) linked to clinical nursing practice.

It was illustrated from the current work that only about one-quarter of students (25.8%) who were diagnosed as IBS cases using Rome III Criteria were previously diagnosed by a physician. Studies from Iran (6) and from Bangladesh (26) found that 37.7 and 35% of IBS cases had visited physicians, respectively.

The reported associations that are corroborated in the current study are female gender, morbid anxiety, living in a school dormitory, emotional stress, and higher academic year. Females were about three times more prone to IBS compared than males. This finding appears to support the results of previous fairly strong associations between female gender and IBS (2, 4, 6, 7, 11, 13, 15, 20, 22, 27, 28). Results of a systematic review from Iran, 2012 (7), showed that more than half of the reviewed studies demonstrated that the prevalence of IBS had statistically a significant correlation with the female gender.

Concerning age and academic year, the present study revealed that the prevalence of IBS was higher among older students and those from higher academic levels (especially fifth and sixth year students and interns). This may be due to increased study and work stressors during clinical years and internship. The cause of the lower rate among the fourth year students may be attributed to lower clinical load during this year. Chu et al. (15) reported that higher grade undergraduates had more risks for FBD than lower grade students, especially among medical students. On the other hand, Payne et al. 2004 (29) showed that there was no relation between age and prevalence of IBS. The 2012 study from Ontario, Canada, (16) also found that there were no significant differences between preclinical and clerkship students regarding IBS prevalence. Jahangiri et al. (7) conducted a systematic review in Iran, which showed that IBS was more prevalent in the first and second year compared to fourth and fifth year medical students.

Students with a positive family history of IBS in the current study were about two times more prone to it compared to others. This agrees with results of a family-based case–control research conducted in the United States, which confirmed IBS familial clustering and highlighted that family history is a known predictor of IBS (30).

Mansour-Ghanaei et al. (6) reported that students living at a distance from their families had significantly higher rates of IBS compared to others. This concurs with results of the present study.

Studies have reported that IBS is associated with emotional and psychological stress (20, 31). In the present study, emotional stress was one of the predictors of IBS. Al-Turki et al. 2011 (8) found that psychological stress was much higher among students who had IBS in King Saud University, Riyadh, Saudi Arabia.

Regarding anxiety, the current study revealed that IBS prevalence was higher among participants with morbid and borderline anxiety compared to others. In logistic regression analysis, morbid anxiety was the second predictor of IBS. Naeem et al. (20) conducted a study to determine the prevalence and associated factors of IBS among medical students in Karachi, Pakistan. They found that the psychological symptoms of anxiety were encountered among 55.8% of participants with IBS. Sugaya et al. 2008 (3) reported that individuals with IBS in Japan had higher scores on the HADS than the control group (12). Similar results were also reported from many other studies (2, 5, 11, 17, 26, 30–32). These findings stress the need to retain a focus on psychological as well as physical factors for the management of IBS (5).

In the current study, students with morbid depression had a higher prevalence (41.9%) of IBS compared to those with borderline depression (29.5%) or normal students (31.5%). However, there was no statistically significant difference. Tan et al. (23) reported that 24.4% of students complaining of IBS have depression.

Regarding sleep, the present study showed that the students who sleep less than 8 h/day had a slightly higher prevalence of IBS compared to others. The study of Al-Turki et al. (8) showed that students with IBS had a significantly higher rate of insomnia compared to others. Okami et al. (12) found that sleep disorders and the time spent sitting were also higher in males with IBS. On the other hand, the Canadian study (16) reported that medical students on overnight call was not associated with the development of IBS.

The current work revealed that IBS prevalence was much higher among participants with food hypersensitivity; see Carroccio et al. (33). On the other hand, our results showed that there were no statistically significant differences between the intake of different food items and the prevalence of IBS, which agrees with the 2011 study among Korean medical students (4). On contrary, Okami et al. (12) illustrated that there was a difference between food product intakes by those with IBS compared to others. The study of Al-Turki et al. (8) also showed that a dietary factor was responsible for 15.5% of IBS.

Regarding habits, the results of the present study showed that IBS prevalence was higher (37.3%) among students who did not practice physical exercise compared to others (26.1%). Kim et al. (34) reported a higher prevalence of IBS in those who did less exercise. Dong et al. (2) found that low exercise levels indicated a high risk for IBS among Chinese university students.

The current study shows an insignificant relationship between smoking and IBS, which agrees with the results of Chirila et al. (35).

## Conclusion

The study illustrated a high prevalence (31.8%) of IBS among medical students and interns in Jeddah. A high rate of anxiety and depression also prevailed. Female gender, morbid anxiety, presence of emotional stress, living away from the family in a school dormitory, and higher academic year were the main predictors of IBS. Screening for IBS and psychological problems is recommended. Stress management courses are required to enable students to cope with different stressors during their medical studies and work.

## References

[1] Gholamrezaei A, Zolfaghari B, Farajzadegan Z, Nemati K, Daghaghzadeh H, Tavakkoli H (2011). Linguistic validation of the Irritable Bowel Syndrome-Quality of Life Questionnaire for Iranian patients. Acta Med Iran.

[2] Dong YY, Zuo XL, Li CQ, Yu YB, Zhao QJ, Li YQ (2010). Prevalence of irritable bowel syndrome in Chinese college and university students assessed using Rome III criteria. World J Gastroenterol.

[3] Sugaya N, Nomura S (2008). Relationship between cognitive appraisals of symptoms and negative mood for subtypes of irritable bowel syndrome. Biopsychosoc Med.

[4] Jung HJ, Park MI, Moon W, Park SJ, Kim HH, Noh EJ (2011). Are food constituents relevant to the irritable bowel syndrome in young adults? – A Rome III based prevalence study of the Korean medical students. J Neurogastroenterol Motil.

[5] Jones R, Latinovic R, Charlton J, Gulliford M (2006). Physical and psychological co-morbidity in irritable bowel syndrome: a matched cohort study using the General Practice Research Database. Aliment Pharmacol Ther.

[6] Mansour-Ghanaei F, Fallah MS, Heidarzadeh A, Jafarshad R, Joukar F, Rezvan-Ghasemipour (2011). Prevalence and characteristics of irritable bowel syndrome (IBS) amongst medical students of Gilan Northern Province of Iran. MEJDD.

[7] Jahangiri P, Jazi MS, Keshteli AH, Sadeghpour S, Amini E, Adibi P (2012). Irritable Bowel Syndrome in Iran: SEPAHAN systematic review No. 1. Int J Prev Med.

[8] Al-Turki Y, Aljulifi YA, Al Murayshid A, Al Omaish HR, Al Daghiri KS, Al Seleemi AY (2011). Prevalence of Irritable Bowel Syndrome among Students in King Saud University, Riyadh, Saudi Arabia. World Fam Med J.

[9] Jafri W, Yakoob J, Jafri N, Islam M, Ali QM (2005). Frequency of irritable bowel syndrome in college students. J Ayub Med Coll Abbottabad.

[10] Mousavinasab SM, Gorganinezhad-Moshiri M, Saberifirouzi M, Dehbozorgi G, Mehrabani D (2007). Personality characteristics and irritable bowel syndrome in Shiraz, southern Iran. Saudi J Gastroenterol.

[11] Abdulmajeed A, Rabab MA, Sliem HA, Hebatallah NE (2011). Pattern of irritable bowel syndrome and its impact on quality of life in primary health care center attendees, Suez governorate, Egypt. Pan Afr Med J.

[12] Alhazmi AH (2011). Irritable bowel syndrome in secondary school male students in AlJouf Province, north of Saudi Arabia. J Pak Med Assoc.

[13] Okami Y, Kato T, Nin G, Harada K, Aoi W, Wada S (2011). Lifestyle and psychological factors related to irritable bowel syndrome in nursing and medical school students. J Gastroenterol.

[14] Jimenez C, Navia-Osorio PM, Diaz CV (2010). Stress and health in novice and experienced nursing students. J Adv Nurs.

[15] Chu L, Zhou H, Lü B, Li M, Chen MY (2012). [An epidemiological study of functional bowel disorders in Zhejiang college students and its relationship with psychological factors]. Zhonghua Nei Ke Za Zhi.

[16] Wells M, Roth L, McWilliam M, Thompson K, Chande N (2012). A cross-sectional study of the association between overnight call and irritable bowel syndrome in medical students. Can J Gastroenterol.

[17] Shen L, Kong H, Hou X (2009). Prevalence of irritable bowel syndrome and its relationship with psychological stress status in Chinese university students. J Gastroenterol Hepatol.

[18] Wang W (2012). Clinical Epidemiology-Basic Principles and Practical Applications.

[19] Zigmond AS, Snaith RP (1983). The hospital anxiety and depression scale. Acta Psychiatr Scand.

[20] Naeem SS, Siddiqui EU, Kazi AN, Memon AA, Khan ST, Ahmed B (2012). Prevalence and factors associated with irritable bowel syndrome among medical students of Karachi, Pakistan: a cross-sectional study. BMC Res Notes.

[21] Longstreth GF, Thompson WG, Chey WD, Houghton LA, Mearin F, Spiller RC (2006). Functional bowel disorders. Gastroenterology.

[22] Hungin AP, Whorwell PJ, Tack J, Mearin F (2003). The prevalence, patterns and impact of irritable bowel syndrome: an international survey of 40,000 subjects. Aliment Pharmacol Ther.

[23] Tan YM, Goh KL, Muhidayah R, Ooi CL, Salem O (2003). Prevalence of irritable bowel syndrome in young adult Malaysians: a survey among medical students. J Gastroenterol Hepatol.

[24] Kang JY (2005). Systematic review: the influence of geography and ethnicity in irritable bowel syndrome. Aliment Pharmacol Ther.

[25] Hazlett-Stevens H, Craske MG, Mayer EA, Chang L, Naliboff BD (2003). Prevalence of irritable bowel syndrome among university students: the roles of worry, neuroticism, anxiety sensitivity and visceral anxiety. J Psychosom Res.

[26] Masud MA, Hasan M, Khan AK (2001). Irritable bowel syndrome in a rural community in Bangladesh: prevalence, symptoms pattern, and health care seeking behavior. Am J Gastroenterol.

[27] Song SW, Park SJ, Kim SH, Kang SG (2012). Relationship between irritable bowel syndrome, worry and stress in adolescent girls. J Korean Med Sci.

[28] Adeyemo MA, Chang L (2008). New treatments for irritable bowel syndrome in women. Womens Health. (Lond Engl).

[29] Payne S (2004). Sex, gender, and irritable bowel syndrome: making the connections. Gend Med.

[30] Saito YA, Petersen GM, Larson JJ, Atkinson EJ, Fridley BL, de Andrade M (2010). Familial aggregation of irritable bowel syndrome: a family case-control study. Am J Gastroenterol.

[31] Savas LS, White DL, Wieman M, Daci K, Fitzgerald S, Laday Smith S (2009). Irritable bowel syndrome and dyspepsia among women veterans: prevalence and association with psychological distress. Aliment Pharmacol Ther.

[32] Gros DF, Antony MM, McCabe RE, Swinson RP (2009). Frequency and severity of the symptoms of irritable bowel syndrome across the anxiety disorders and depression. J Anxiety Disord.

[33] Carroccio A, Brusca I, Mansueto P, Soresi M, D'Alcamo A, Ambrosiano G (2011). Fecal assays detect hypersensitivity to cow's milk protein and gluten in adults with irritable bowel syndrome. Clin Gastroenterol Hepatol.

[34] Kim YJ, Ban DJ (2005). Prevalence of irritable bowel syndrome, influence of lifestyle factors and bowel habits in Korean college students. Int J Nurs Stud.

[35] Chirila I, Petrariu FD, Ciortescu I, Mihai C, Drug VL (2012). Diet and irritable bowel syndrome. J Gastrointestin Liver Dis.

